# Supplementary material for: MicroRNA Signatures Associated with Bronchopulmonary Dysplasia Severity in Tracheal Aspirates of Preterm Infants
Source: Biomedicines. 2021 Mar 5;9(3):257. doi: 10.3390/biomedicines9030257 (PMC8000397; doi:10.3390/biomedicines9030257)
Supplement: Supplementary file 1 [file biomedicines-09-00257-s001.pdf]

biomedicines

MDPI

Supplementary Material

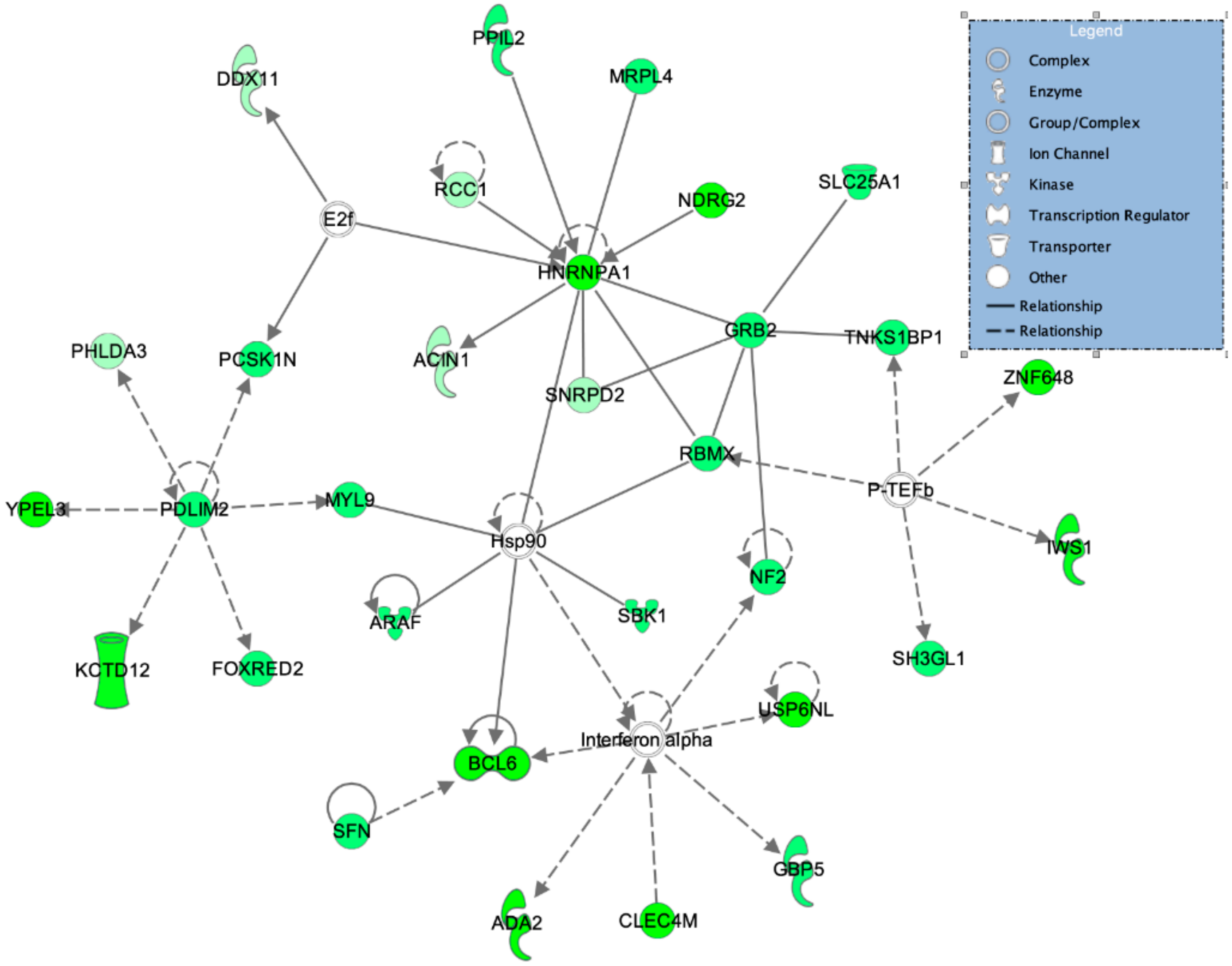

Figure 1. Respiratory disease, cancer, organismal injury and abnormalities network.

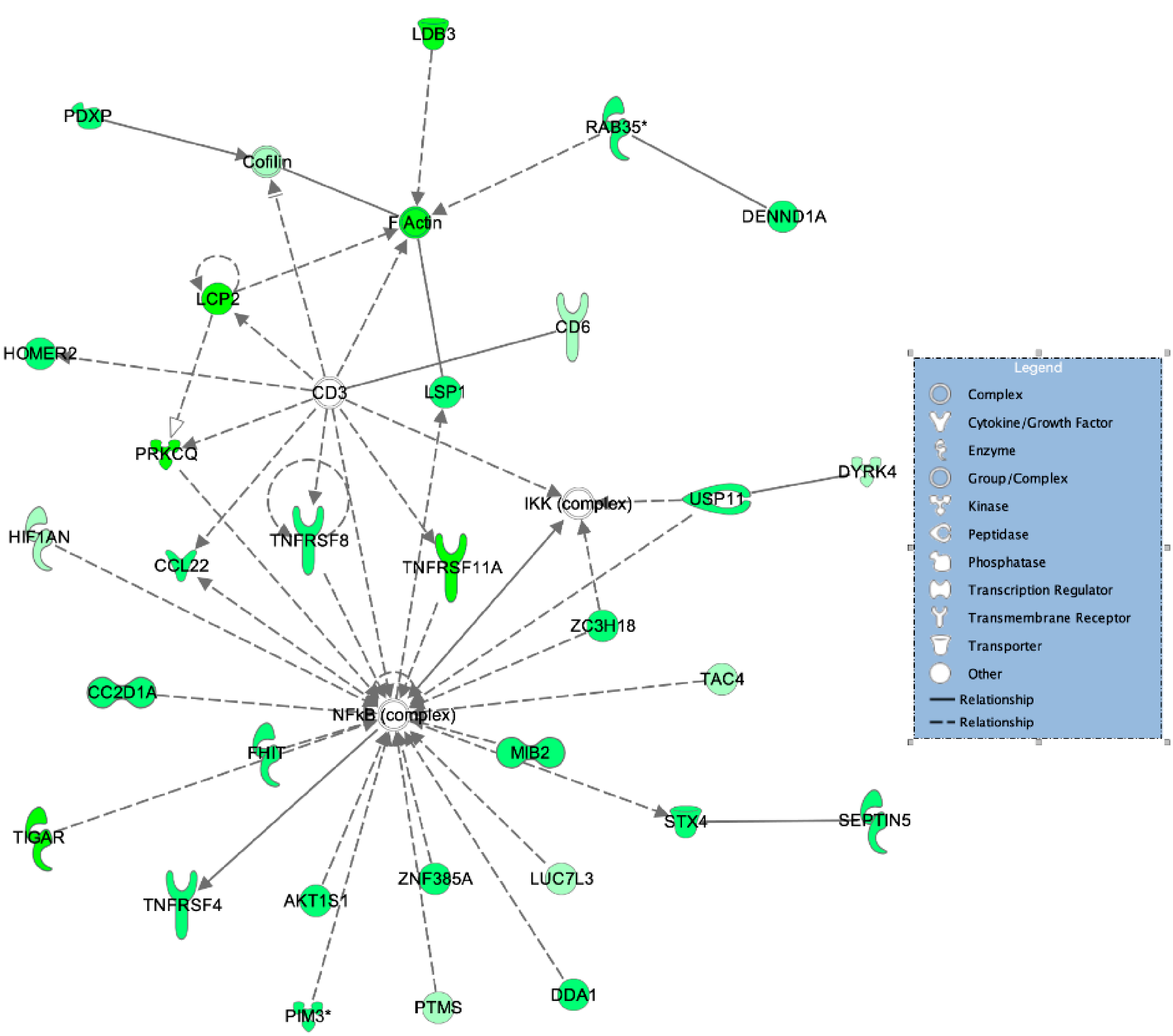

Figure 2. Cellular movement, immune cell trafficking, immunological disease network.
